# Effects of mind–body therapies on sleep quality in perinatal women: a systematic review and meta-analysis of randomized controlled trials

**DOI:** 10.3389/fpubh.2026.1857655

**Published:** 2026-06-17

**Authors:** Longkang Guo, Yuchao Li, Lintao Suo, Jingyuan Yang, Xuehaiyue Lv, Hengyi Song, Wei Han

**Affiliations:** 1 Graduate Education College, Shandong Sport University, Jinan, Shandong, China; 2 Sports Department, University of Electronic Science and Technology of China, Chengdu, China; 3 College of Competitive Sports, Shandong Sport University, Rizhao, China

**Keywords:** meta-analysis, mind–body therapies, perinatal women, sleep quality, systematic review

## Abstract

**Background:**

Sleep quality often worsens in perinatal women because of physiological, endocrine, and psychosocial changes. This may increase the risk of maternal and infant complications, making it a public health concern. Because pharmacological treatments may pose safety risks, mind–body therapies have gained attention as a safe non-pharmacological option. However, current evidence mainly focuses on single intervention types, and their overall effect on sleep quality in perinatal women remains unclear.

**Methods:**

We systematically searched seven databases—PubMed, Web of Science, Embase, the Cochrane Library, Scopus, EBSCO, and ScienceDirect—and other sources up to January 20, 2026, to identify randomized controlled trials examining the effects of mind–body therapies on sleep quality in perinatal women. Two reviewers independently screened the literature, extracted the data, and assessed the risk of bias using the Cochrane Risk of Bias 2 (ROB2) tool. Meta-analysis was performed in Stata 17 using a random-effects model. Subgroup, sensitivity, and publication bias analyses were also conducted. In addition, the certainty of evidence for the primary outcome was assessed using the Grading of Recommendations Assessment, Development and Evaluation (GRADE) approach.

**Results:**

A total of 13 randomized controlled trials involving 782 participants were included. The main interventions were mindfulness, Pilates, and yoga. Meta-analysis showed that, compared with controls, mind–body therapies significantly improved sleep quality in perinatal women (MD = −2.63, 95% CI −3.36 to −1.90). However, substantial heterogeneity was observed across studies (I^2^ = 89.0%, *P* < 0.001). Subgroup analyses showed significant between-group differences by country (*P* < 0.05), intervention duration (*P* < 0.01), and session length (*P* < 0.05), suggesting that these factors may partly explain heterogeneity. Sensitivity analysis and risk-of-bias assessment suggested that the results were statistically robust, and no significant publication bias was detected (*P* = 0.900). According to GRADE, the certainty of evidence for the primary outcome was rated as low.

**Conclusions:**

Mind–body therapies effectively improve sleep quality in perinatal women and may serve as a useful adjunct in clinical sleep health management. However, the certainty of evidence was rated as low according to GRADE; therefore, these findings should be interpreted cautiously and further confirmed by high-quality studies.

**Systematic review registration:**

https://www.crd.york.ac.uk/PROSPERO/view/CRD420261290532, identifier: CRD420261290532.

## 1 Introduction

The perinatal period is a unique physiological stage for women and is generally defined as the time from the onset of pregnancy to one year postpartum (1–3). During this period, changes in physiological structure, marked fluctuations in endocrine hormone levels, and multiple psychosocial influences together contribute to a significant decline in sleep quality (4). Poor sleep quality is commonly manifested as difficulty falling asleep (sleep onset latency >30 min in adults), sleep maintenance disturbance (≥2 nocturnal awakenings per night), early morning awakening, subjective decline in sleep quality, and reduced total sleep time (<6.5 h), and may be accompanied by daytime functional impairments such as fatigue, reduced energy, daytime sleepiness, and impaired attention or memory (5, 6). Studies have shown that 88.8% of pregnant women experience sleep disturbances during pregnancy, including insomnia and reduced daytime alertness (7). The prevalence of poor sleep quality is about 25% in early pregnancy. This proportion gradually increases as pregnancy progresses and may reach 75% in late pregnancy. The severity of sleep impairment is also positively associated with gestational age (8). These sleep problems are not limited to pregnancy. After childbirth, many women still experience shorter nighttime sleep, more frequent awakenings, and reduced sleep efficiency (9).

Poor sleep quality during the perinatal period not only directly impairs women's physical and mental health, but may also lead to a range of maternal and neonatal complications, imposing a substantial public health burden (10). Studies have shown that poor sleep quality during the perinatal period may significantly increase the risk of restless legs syndrome, gestational hypertension, preeclampsia, and perinatal depression (11). Yang et al. further showed that poor sleep quality is closely associated with adverse pregnancy outcomes, including gestational diabetes mellitus and preterm birth (12). More importantly, poor sleep quality during the perinatal period may affect normal fetal development by altering the intrauterine environment, thereby leading to adverse fetal outcomes (13). Taken together, sleep health in perinatal women has become an important public health issue that requires urgent attention and effective intervention.

At present, interventions to improve sleep quality in clinical practice mainly fall into two categories: pharmacological and non-pharmacological treatments. Although drug therapy can relieve sleep disturbances quickly, studies have shown that it may cause potential adverse effects in both mothers and fetuses, and its safety cannot be fully assured (14). This has limited its clinical use in the perinatal population. In contrast, mind-body interventions are safe and feasible non-pharmacological approaches that have garnered increasing attention in the field of sleep health in recent years. These interventions mainly include yoga, Pilates, and mindfulness. Unlike simple physical exercise, they place greater emphasis on regulating the interaction among the brain, mind, body, and behavior through specific practices, thereby improving sleep quality more effectively (15). A meta-analysis of 27 randomized controlled trials showed that mind–body therapies, including Pilates, yoga, qigong, and tai chi, can effectively reduce sleep disturbances and depression (16). In addition, another meta-analysis found that mind–body therapies had positive effects on bone mineral density, sleep quality, anxiety, depression, and fatigue in perimenopausal and postmenopausal women (17).

Although mind-body interventions have shown clear benefits in improving sleep quality in specific populations, the current evidence still has important limitations. Most existing studies have focused on older adults and perimenopausal women. Research on mind–body therapies for sleep quality in perinatal women remains limited. In addition, most available evidence has examined only a single type of intervention, such as yoga or Pilates alone (18, 19), To date, no study has systematically and comprehensively examined the overall effect of mind–body therapies, as a broad category of intervention, on sleep quality in perinatal women. In addition, existing meta-analyses on sleep interventions for perinatal women have mostly used the standardized mean difference (SMD) as the main pooled effect size when combining outcomes. Few studies have used a common assessment tool and adopted the mean difference (MD), which is more directly clinically interpretable. This has, to some extent, limited the clinical applicability and practical value of their findings (20, 21).

In light of these gaps, this study aimed to systematically evaluate the overall effectiveness of mind–body therapies, as a broad category of intervention, in improving sleep quality in perinatal women. To address the methodological limitations of previous studies, we used the MD as the pooled effect size, given that all included studies assessed outcomes using the total Pittsburgh Sleep Quality Index (PSQI) score. This approach was intended to improve the clinical interpretability of the findings and provide a more direct presentation of the results under a uniform measurement framework. We hope that this study will offer a clearer and more reliable evidence summary for clinicians, policymakers, and perinatal women, thereby informing clinical practice, optimizing sleep health interventions during the perinatal period, and guiding future high-quality research.

## 2 Materials and methods

This study strictly complied with the PRISMA 2020 statement and the Cochrane Handbook for conducting systematic reviews and meta-analyses (22). The protocol was registered in the International Prospective Register of Systematic Reviews (PROSPERO: CRD420261290532)(https://www.crd.york.ac.uk/PROSPERO/view/CRD420261290532).

### 2.1 Literature search strategy

We searched seven databases: PubMed, Web of Science, EBSCO, the Cochrane Library, Scopus, Embase, and ScienceDirect. The search covered the period from database inception to January 20, 2026. A combination of subject terms and free-text terms was used to identify randomized controlled trials on the effects of mind–body therapies on sleep quality in perinatal women. PubMed was used as an example, and the detailed search strategy is shown in Table 1.

**Table 1:** PubMed search strategy.

Steps	Search strategies
#1	[“Exercise” (Mesh)] OR [“Mind-Body Therapies” (Mesh)] OR (mind-body exercise) OR (yoga) OR (pilates) OR (tai chi) OR (mindfulness) OR (meditation) OR (Qi gong) OR (Mind-Body Practice) OR (Baduanjin) OR (Wuqinxi) OR (Mind Body Intervention)
#2	[“Perinatal Care” (Mesh)] OR [“Pregnancy” (Mesh)] OR [“Prenatal Care” (Mesh)] OR [“Postpartum Period” (Mesh)] OR [“Postnatal Care” (Mesh)] OR [“Peripartum Period” (Mesh)] OR (perinatal) OR (antenatal) OR (postnatal) OR (post-natal) OR (postpartum) OR (post-partum) OR (pregnant) OR (prenatal) OR (childbearing) OR (peripartum) OR (puerperium) OR (antepartum) OR (parturition) OR [childbirth (Title/Abstract)] OR (gestation) OR (pre-natal)
#3	[“Sleep” (Mesh)] OR [“Sleep Initiation and Maintenance Disorders” (Mesh)] OR [“Sleep Quality” (Mesh)] OR [“Dyssomnias” (Mesh)] OR [(sleeping) OR (poor sleep) OR (sleep problem) OR (sleep disturbance) OR (dysomnia) OR (sleep initiation)] OR (sleep maintenance) OR (sleep disorder) OR (sleep restriction) OR (sleep hygiene) OR (sleep latency) OR (sleep duration) OR (sleep efficiency) OR (daytime dysfunction) OR (sleep outcome) OR (sleep characteristics) OR (sleep quantity) OR (difficulty falling asleep) OR (restless sleep) OR (awakenings) OR (snoring) OR (diurnal sleep) OR (diurnal tiredness) OR (sleepiness) OR (sleep dysfunction) OR (sleep health) OR (sleep time) OR (sleep pattern) OR (sleep parameters)
#4	[randomized controlled trial (Title/Abstract)] OR [randomized (Title/Abstract)] OR [placebo (Title/Abstract)]
#5	#1 AND #2 AND #3 AND #4

### 2.2 Inclusion and exclusion criteria for literature

The inclusion and exclusion criteria of this study were established based on the PICOS framework, covering population, intervention, comparison, outcomes, and study design (23).

#### 2.2.1 Inclusion criteria for literature

(1) **Population (P):** Eligible participants were perinatal women aged 18 years or older. In this study, perinatal women were defined as women from the onset of pregnancy to one year postpartum (1–3). Participants were required to have no pregnancy complications, such as gestational hypertension, diabetes, preeclampsia, or a history of preterm birth; no severe physical illnesses, such as cardiovascular or musculoskeletal disorders; and no psychiatric disorders, such as major depression or schizophrenia.

(2) **Intervention (I):** Participants in the intervention group received mind–body therapy interventions, mainly including yoga, Pilates, and mindfulness (24).

(3) **Comparison (C):** The control group received no intervention or usual care.

(4) **Outcome (O):** To reduce bias arising from heterogeneity in outcome measures, only studies using the total PSQI score as the outcome were included. The PSQI is a commonly used subjective sleep quality assessment tool in clinical and research settings. It quantitatively evaluates an individual's sleep status across seven components: subjective sleep quality, sleep latency, sleep duration, habitual sleep efficiency, sleep disturbances, use of sleep medication, and daytime dysfunction. Each component is scored from 0 to 3, with a total score ranging from 0 to 21; higher scores indicate poorer sleep quality. Therefore, in the present study, a decrease in the PSQI total score represents an improvement in sleep quality (25). The MD used in the subsequent meta-analysis reflects the mean difference in PSQI total scores between the intervention and control groups. A negative MD indicates that the intervention group had a lower PSQI total score than the control group, suggesting a greater improvement in sleep quality.

(5) **Study design (S):** Only randomized controlled trials were included, as this design allows for stricter control of confounding factors and improves the rigor and reliability of the findings (26).

#### 2.2.2 Literature exclusion criteria

(1) Studies involving women with severe physical illness, a history of psychiatric disorders, or pregnancy complications were excluded.
(2) Studies without a control group were excluded.
(3) Reviews, case reports, clinical reports, conference abstracts, and other non-randomized studies were excluded.
(4) Studies with unclear outcome measures or incomplete data were excluded. For example, studies that did not report the PSQI, or provided data for only one time point, either pre-intervention or post-intervention, were not included.

### 2.3 Literature screening and data extraction

Study selection and data extraction were performed independently by two reviewers (Yuchao Li and Lintao Suo). Both reviewers strictly followed the predefined inclusion and exclusion criteria to screen, organize, and preliminarily process the studies. The results were then cross-checked to ensure consistency. Any disagreements during screening or processing were resolved by a third reviewer (Jingyuan Yang) through discussion until consensus was reached, after which data extraction proceeded. The extracted data covered key study characteristics, including publication year, mean age and gestational age of participants, sample size, type of intervention, intervention duration in weeks, intervention frequency, and session length.

### 2.4 Risk assessment of bias

Two reviewers (Yuchao Li and Lintao Suo) independently assessed the risk of bias of the included studies using the Cochrane Risk of Bias 2 (ROB 2) tool. This tool covers five core domains: (a) randomization process, (b) deviations from intended interventions, (c) missing outcome data, (d) measurement of the outcome, and (e) selection of the reported result. It also includes an overall risk-of-bias judgment (Overall Bias) (27). The risk of bias was classified as follows. Each core domain was rated as having low risk, some concerns, or high risk of bias. The overall risk of bias for each study was then determined based on the ratings across these domains. A study was judged as low risk if all key domains were rated as low risk. It was judged as some concerns if one or more domains raised some concerns, but none were rated as high risk. It was judged as high risk if at least one key domain was rated as high risk. Any disagreement between the two reviewers was checked again by a third reviewer (Jingyuan Yang) until a final consensus was reached.

### 2.5 Statistical analysis

Data synthesis and meta-analysis were performed using Stata software (version 17; StataCorp, College Station, TX, USA) (28). Because all included studies used the total PSQI score as the outcome measure, the MD with its 95% confidence interval (CI) was used as the pooled effect size (29). Statistical heterogeneity across studies was assessed using both the Cochrane *Q* test and the *I*^2^ statistic, with statistical significance set at *P* < 0.10. Heterogeneity was interpreted as follows: when no significant heterogeneity was detected (*I*^2^ ≤ 50% and *P* ≥ 0.10), a fixed-effect model was used; when significant heterogeneity was present (*I*^2^ >50% and *P* < 0.10), potential sources of heterogeneity were further explored. After excluding the influence of obvious clinical heterogeneity, a random-effects model was used for meta-analysis. For studies with substantial clinical heterogeneity, subgroup analyses and other appropriate methods were applied (30). Sensitivity analyses were also conducted to test the robustness of the main findings. Publication bias was assessed using funnel plot inspection together with Egger's test (31). The certainty of evidence for the primary outcome was assessed using the GRADE approach.

## 3 Results

### 3.1 Results of literature screening

**Figure 1:**
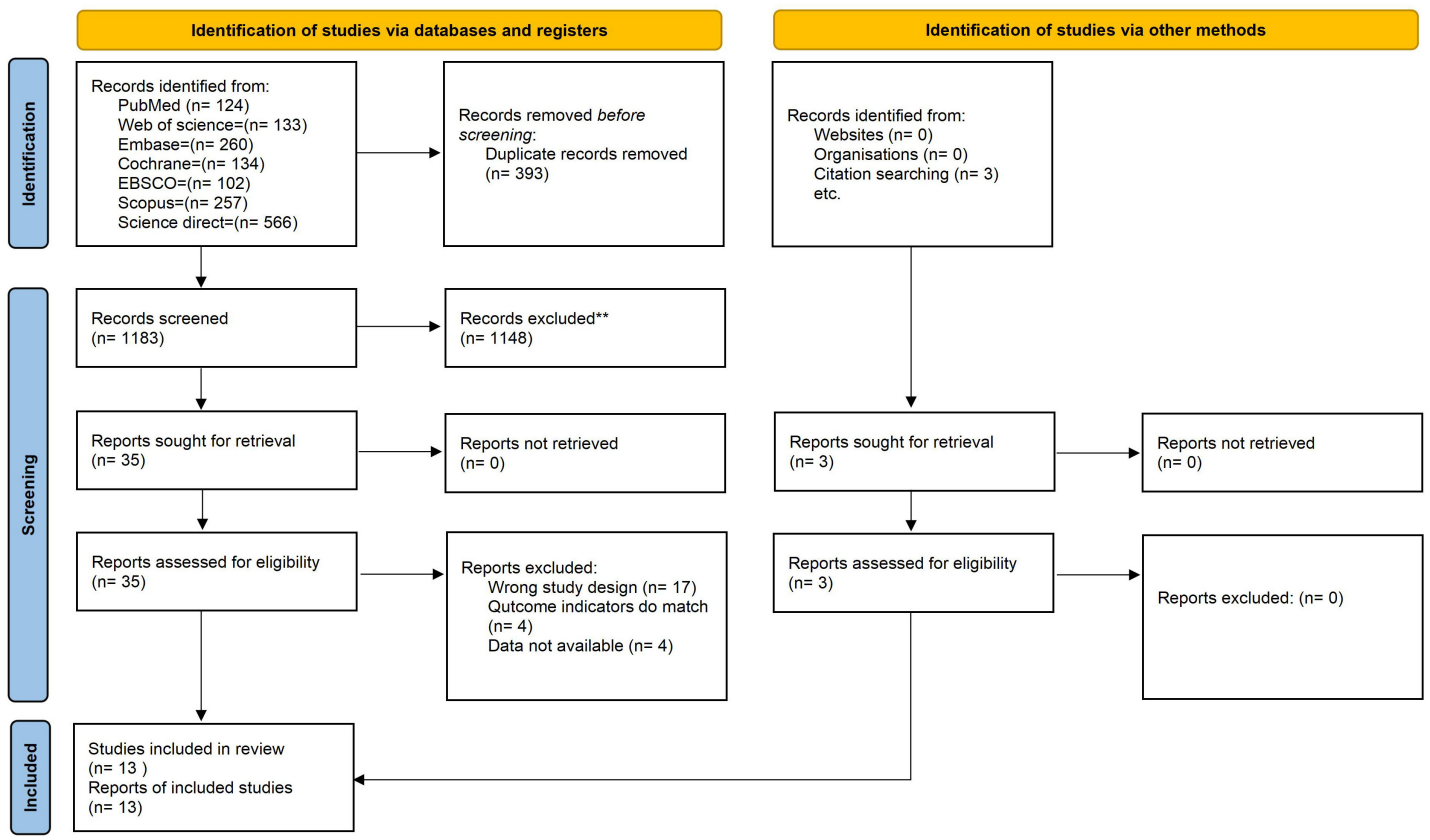
Flow diagram of the study selection process.

This study systematically searched seven databases, including PubMed, Web of Science, Embase, Cochrane Library, Scopus, EBSCO, and ScienceDirect, and identified 1,576 records. In addition, 3 potentially relevant records were identified through citation searching, which was conducted by manually screening the reference lists of the included studies and relevant systematic reviews or meta analyses. Therefore, a total of 1,579 records were initially identified. After 393 duplicates were removed, the titles and abstracts of the remaining 1,186 records were screened. Of these, 1,148 were excluded because they were not relevant to the topic, were not randomized controlled trials, or were not published in English. The full texts of the remaining 38 articles were then assessed for eligibility. Among them, 25 were excluded because of ineligible study design (*n* = 17), unmatched outcome measures (*n* = 4), or missing data (*n* = 4). Ultimately, 13 studies met all inclusion criteria and were included in this systematic review and meta-analysis. The full study selection process is shown in the PRISMA flow diagram (Figure 1).

### 3.2 Basic characteristics of the included studies

A total of 13 randomized controlled trials were included in this study. They were published between 2012 and 2025 and were mainly conducted in the United States (32), Iran (33–39), South Korea (40, 41), China (42, 43) and Indonesia (44). In 3 studies, participants were in the postpartum period [31, 33, 35], whereas in the other 10 studies, all participants were pregnant women. Altogether, 782 participants were included, with 397 in the intervention group and 385 in the control group. The main intervention types were mindfulness, Pilates, and yoga, including 6 mindfulness-based interventions (32, 33, 35, 36, 39, 42), 5 Pilates interventions (34, 37, 38, 40, 41), 2 yoga interventions (43, 44). The intervention duration ranged from 4 to 12 weeks, with 8 weeks being the most common duration; specifically, eight studies adopted an 8-week intervention program (33–37, 39, 40, 44). The intervention frequency ranged from once per week to five times per week, and the duration of each session ranged from 10 to 90 min. All included studies used the PSQI as the outcome measure (Table 2).

**Table 2:** Basic characteristics of the included studies.

Study	Country	Sample size	Age	Duration	Intervention category	Frequency	Session length (min)	Gestational age	intervention supervision	Baseline sleep quality	Outcome
Wi et al., (32)	United States	C:22 E:23	C: 33.05 ± 3.92 E: 34.10 ± 4.20	6 weeks	C: Usual education E: Mindfulness	1d/week	20	12 weeks−28 weeks	Unsupervised	mild	PSQI
Ghalgar et al., (36)	Iran	C:44 E:44	C: 27.25 ± 5.16 E: 26.25 ± 5.15	8 weeks	C: Usual Care E: Mindfulness	1d/week	60	Postpartum	Online supervision	severe	PSQI
Shams et al., (39)	Iran	C:34 E:34	18–50	8 weeks	C: Usual care E: Mindfulness	1d/week	60	28 weeks−36 weeks	onsite supervision	mild	PSQI
Mottaghi et al., (38)	Iran	C:27 E:28	C: 24.28 ± 5.85 E: 24.63 ± 5.55	12 weeks	C: Usual care E: Pilates	3d/week	30	20 weeks	onsite supervision	mild	PSQI
Shamabadi et al., (33)	Iran	C:48 E:50	C: 28.08 ± 5.89 E: 28.09 ± 5.44	8 weeks	C: Usual care E: Mindfulness	unclear	90	13 weeks later	Online supervision	mild	PSQI
Mirmohammadali et al., (37)	Iran	C:40 E:40	C: 24.40 ± 3.65 E: 24.60 ± 3.65	8 weeks	C: No intervention E: Pilates	5d/week	30	Postpartum	Unsupervised	severe	PSQI
Qi et al., (42)	China	C:25 E:27	C: 28.69 ± 3.49 E: 28.69 ± 3.66	4 weeks	C: Usual care E: Mindfulness	1d/week	40–50	28weeks −36weeks	Online supervision	severe	PSQI
Kim et al., (41)	South Korea	C:8 E:8	C: 38.14 ± 1.39 E: 39.71 ± 2.01	12 weeks	C: No intervention E: Pilates	2d/week	50	24weeks −28weeks	Online supervision	severe	PSQI
Hyun et al., (40)	South Korea	C:7 E:7	C: 34.14 ± 3.03 E: 34.14 ± 3.82	8 weeks	C: No intervention E: Pilates	2d/week	50	20weeks −24weeks	Online supervision	severe	PSQI
Shen et al., (43)	China	C:47 E:51	C: 32.80 ± 3.83 E: 33.30 ± 4.19	12 weeks	C: Usual care E: yoga	3d/week	20	16weeks −30weeks	Unsupervised	severe	PSQI
Borghei et al., (35)	Iran	C:14 E:15	unclear	8 weeks	C: Usual care E: Mindfulness	1d/week	120–150	14weeks −24weeks	onsite supervision	severe	PSQI
Kundarti et al., (44)	Indonesia	C:29 E:30	C: 25.10 ± 3.27 E: 23.40 ± 3.27	8 weeks	C: Usual care E: yoga	1d/week	90	20 weeks −32 weeks	onsite supervision	mild	PSQI
Ashrafinia et al., (34)	Iran	C:40 E:40	C: 24.40 ± 3.6 E: 24.60 ± 3.6	8 weeks	C: Clinical nursing E: Pilates	5d/week	30	Postpartum	Unsupervised	severe	PSQI

C, Control group; E, Experimental group; Unclear, Not reported in the original studies; d/week, Several times a week; mild: 5–10 score; severe: >10 score.

### 3.3 Evaluation of literature quality

**Figure 2:**
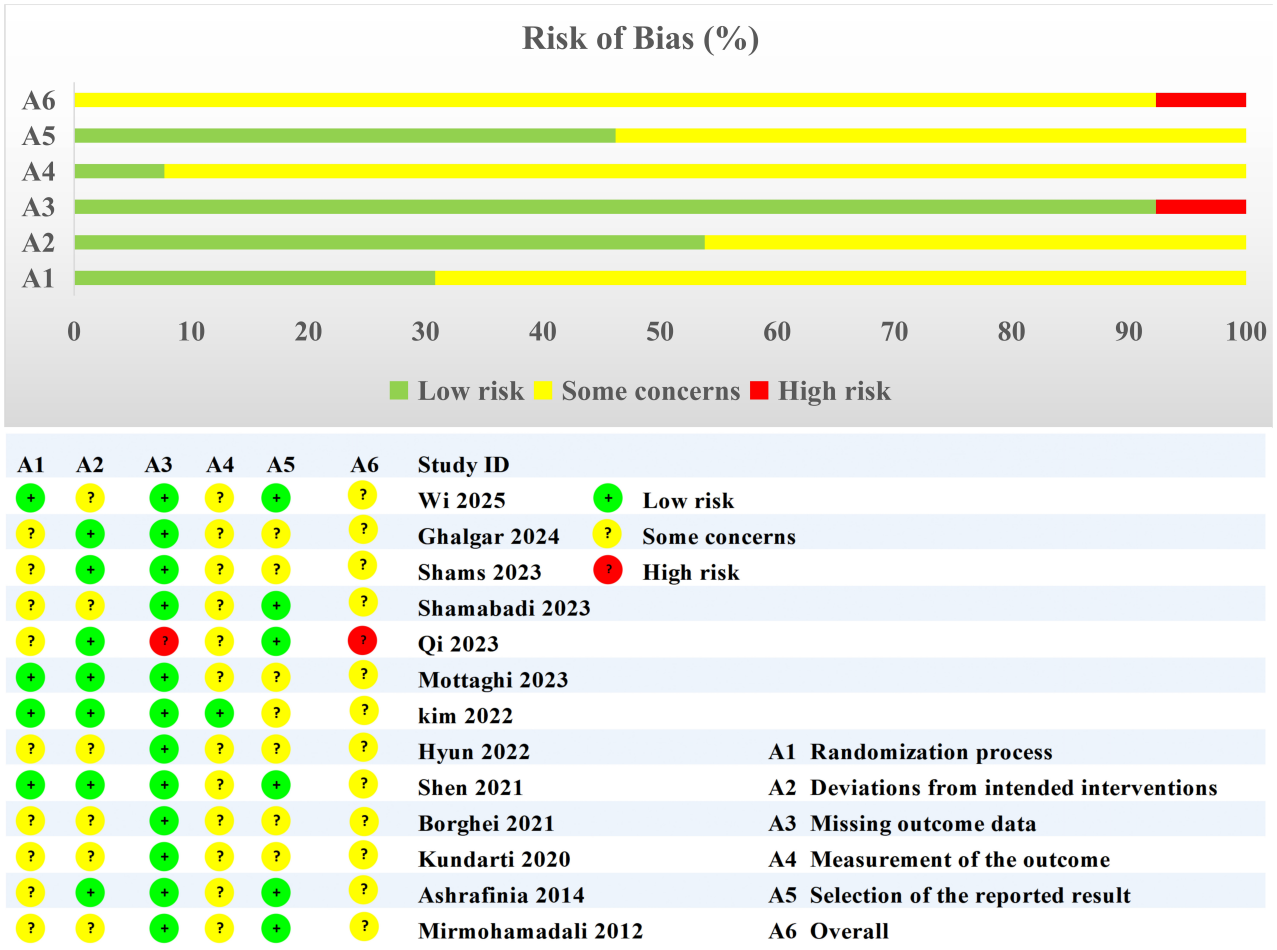
Risk-of-bias assessment of the included studies.

Among the 13 included studies, the overall risk-of-bias assessment showed that 12 studies (92.3%) were rated as having some concerns, and 1 study (7.7%) was rated as high risk. The domain-specific results were as follows. For bias arising from the randomization process, 4 studies were rated as low risk and 9 as some concerns. For bias due to deviations from intended interventions, 6 studies were rated as low risk and 7 as some concerns. For bias due to missing outcome data, 12 studies were rated as low risk and 1 as high risk. For bias in measurement of the outcome, 1 study was rated as low risk and 12 as some concerns. For bias in selection of the reported result, 6 studies were rated as low risk and 7 as some concerns (Figure 2). Overall, the methodological quality of the included studies was relatively low. The ROB2 assessment showed that most studies were rated as having some concerns, and one study was rated as having a high risk of bias. The main concern was related to the randomization process, as nine studies did not provide sufficient information on random sequence generation, allocation concealment, or the detailed implementation of random assignment. These methodological limitations may affect the reliability and certainty of the current evidence.

### 3.4 Meta-analysis results

**Figure 3:**
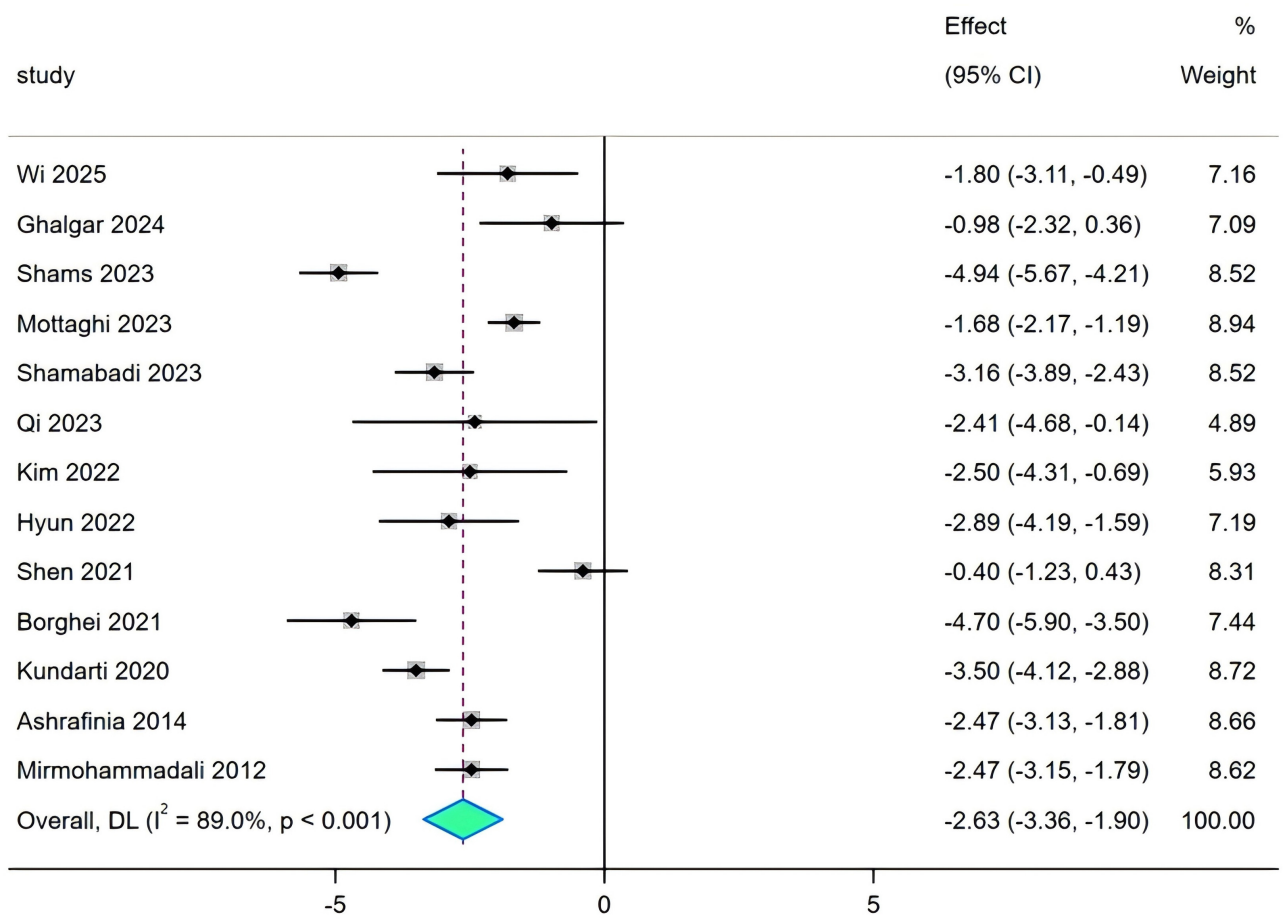
Meta-analysis of the effects of mind-body exercise on sleep quality in perinatal women.

A total of 13 randomized controlled trials were included in the meta-analysis. A random-effects model was used. The results showed that mind–body therapies significantly improved sleep quality in perinatal women (MD = −2.63, 95% CI −3.36 to −1.90), indicating a statistically significant benefit in the intervention group compared with the control group (*P* < 0.001). However, heterogeneity was substantial across studies (*I*^2^ = 89.0%). The detailed results of the meta-analysis are shown in Figure 3.

### 3.5 Subgroup analysis

Because substantial heterogeneity was found in the main meta-analysis, subgroup analyses were further conducted to explore possible sources of heterogeneity and to describe potential differences in intervention effects across study characteristics. We systematically conducted subgroup analyses by stratifying the included studies according to participants' perinatal stage, study country, intervention supervision format, baseline sleep quality, intervention type, intervention duration, intervention frequency, and session duration.

**Figure 4:**
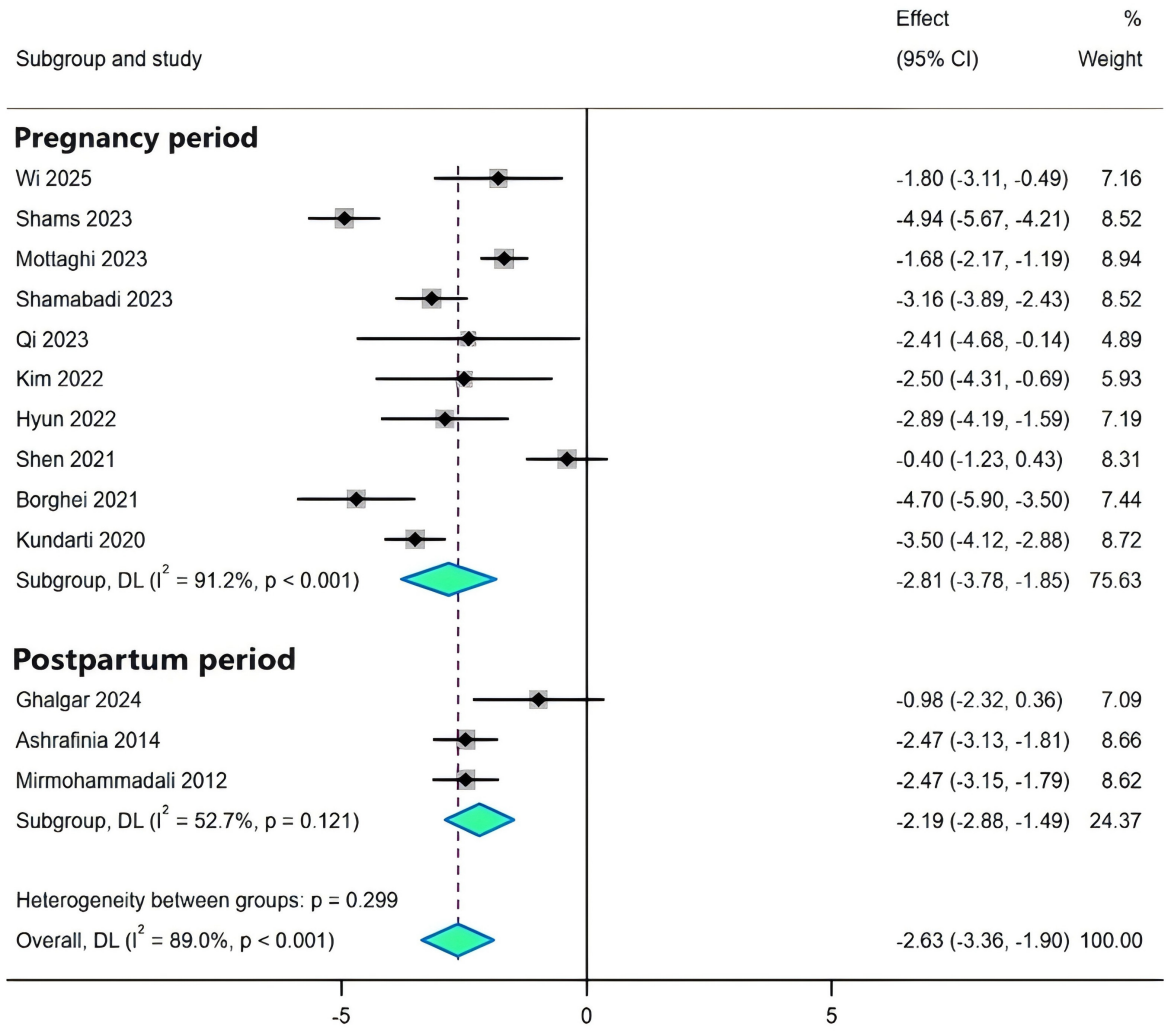
Subgroup analysis by perinatal stage.

Subgroup analysis was conducted according to the perinatal stage of the participants (Figure 4). The test for subgroup differences showed no significant difference between stages (Q = 1.08, df = 1, *P* > 0.05). The pooled results indicated that mind–body therapies significantly improved sleep quality in both pregnant women (MD = −2.81, 95% CI −3.78 to −1.85) and postpartum women (MD = −2.19, 95% CI −2.88 to −1.49).

**Figure 5:**
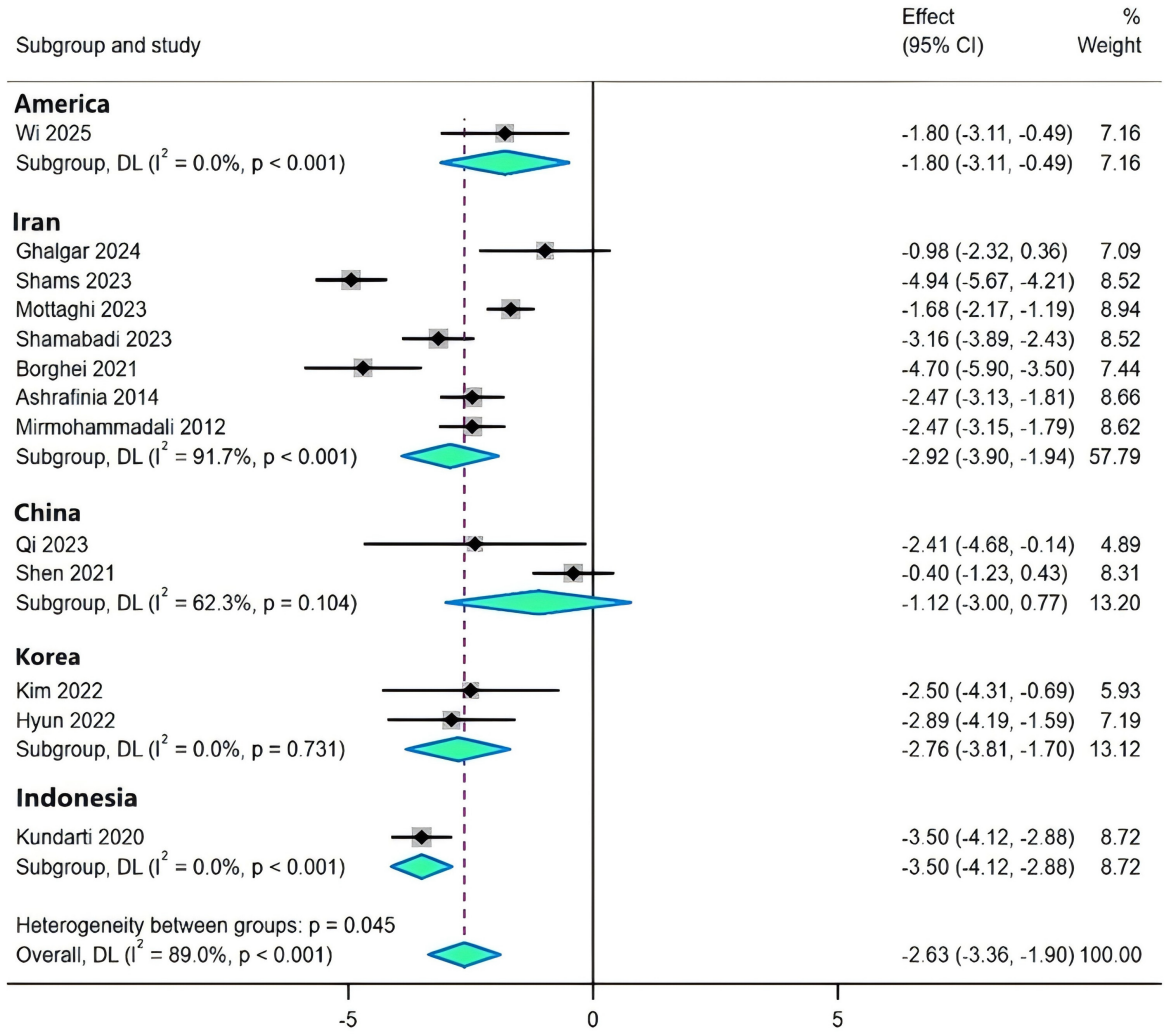
Subgroup analysis by country.

Subgroup analysis was performed by country (Figure 5). The test for subgroup differences showed a significant difference across countries (Q = 9.73, df = 4, *P* < 0.05), suggesting that country may be an important source of the overall heterogeneity. The pooled results showed that mind–body therapies significantly improved sleep quality in perinatal women in studies from the United States (MD = −1.80, 95% CI −3.11 to −0.49), Iran (MD = −2.92, 95% CI −3.90 to −1.94), South Korea (MD = −2.76, 95% CI −3.81 to −1.70), and Indonesia (MD = −3.50, 95% CI −4.12 to −2.88). By contrast, in studies from China, the 95% CI crossed the line of no effect (MD = −1.12, 95% CI −3.00 to 0.77), indicating that the effect of mind–body therapies in this subgroup did not reach statistical significance.

**Figure 6:**
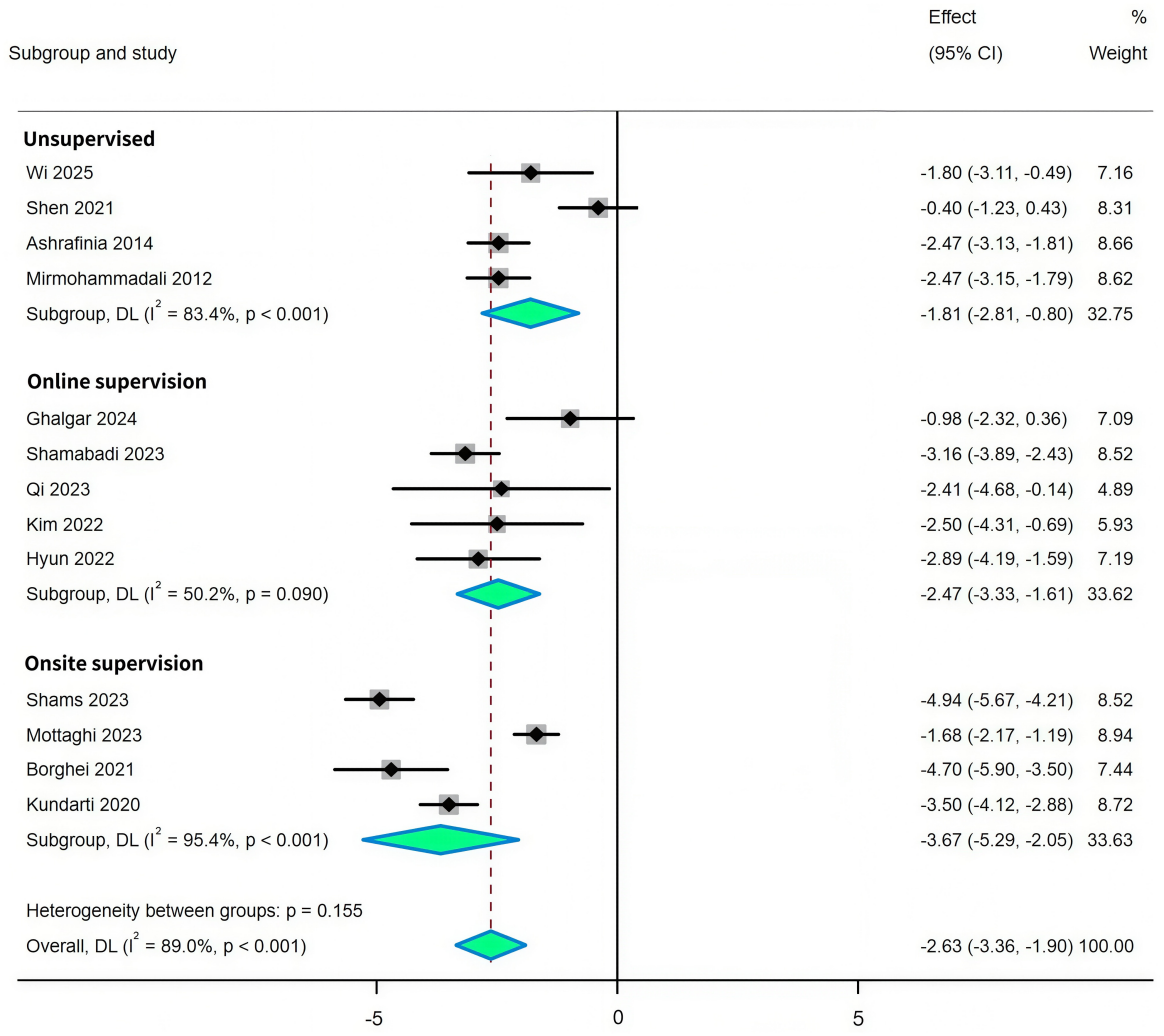
Subgroup analysis by intervention supervision.

Subgroup analysis was performed according to the type of intervention supervision (Figure 6). The test for subgroup differences showed no statistically significant difference in intervention effects across different supervision formats (Q = 3.72, df = 2, *P* > 0.05). The pooled results indicated that non-supervised interventions (MD = −1.81, 95% CI −2.81 to −0.80), online–supervised interventions (MD = −2.47, 95% CI −3.33 to −1.61), and offline-supervised interventions (MD = −3.67, 95% CI−5.29 to−2.05) all significantly improved sleep quality in perinatal women.

**Figure 7:**
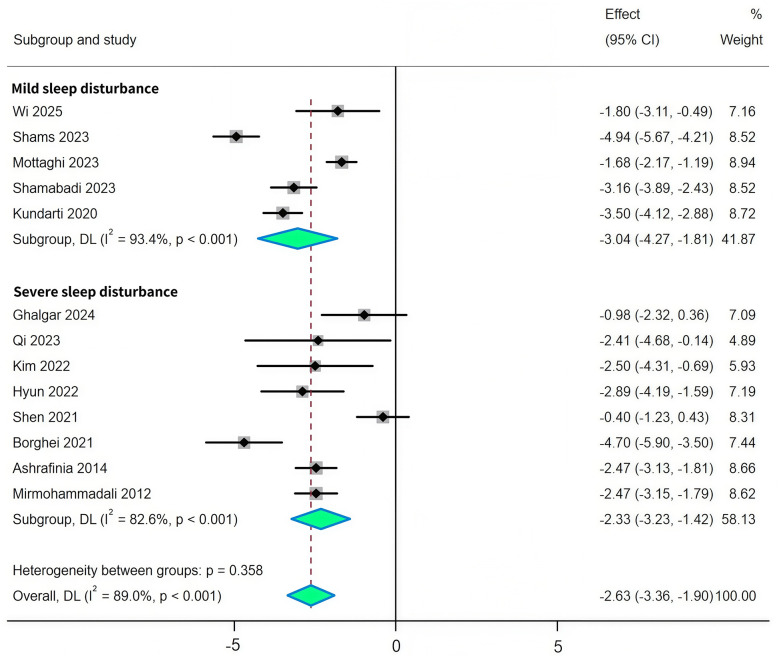
Subgroup analysis by baseline sleep quality.

Subgroup analysis was conducted according to baseline sleep quality (Figure 7). Referring to the classification criteria for PSQI scores used in previous studies, a PSQI score of 5–10 indicates mild sleep disturbance, whereas a score >10 indicates severe sleep disturbance (45). Therefore, the included studies were divided into a mild sleep disturbance group and a severe sleep disturbance group according to baseline PSQI scores. The test for subgroup differences showed no statistically significant difference in intervention effects between different levels of baseline sleep disturbance (Q = 0.84, df = 1, *P* > 0.05). The pooled results showed that mind–body therapies significantly improved sleep quality in perinatal women with either mild sleep disturbance (MD = −2.25, 95% CI −2.72 to −1.79) or severe sleep disturbance (MD = −3.08, 95% CI −4.36 to −1.80).

**Figure 8:**
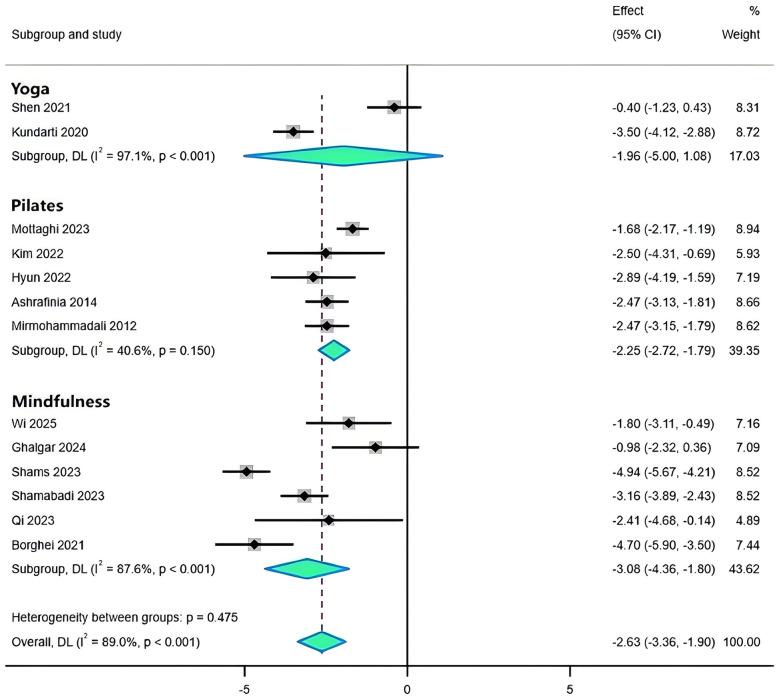
Subgroup analysis by intervention type.

Subgroup analysis was performed by intervention type (Figure 8). The test for subgroup differences showed no significant difference among the intervention types (Q = 1.49, df = 2, *P* > 0.05). The pooled results showed that both Pilates (MD = −2.25, 95% CI −2.72 to −1.79) and mindfulness (MD = −3.08, 95% CI −4.36 to −1.80) significantly improved sleep quality in perinatal women. By contrast, yoga did not show a significant effect on sleep quality in this population (MD = −1.96, 95% CI −5.00 to 1.08).

**Figure 9:**
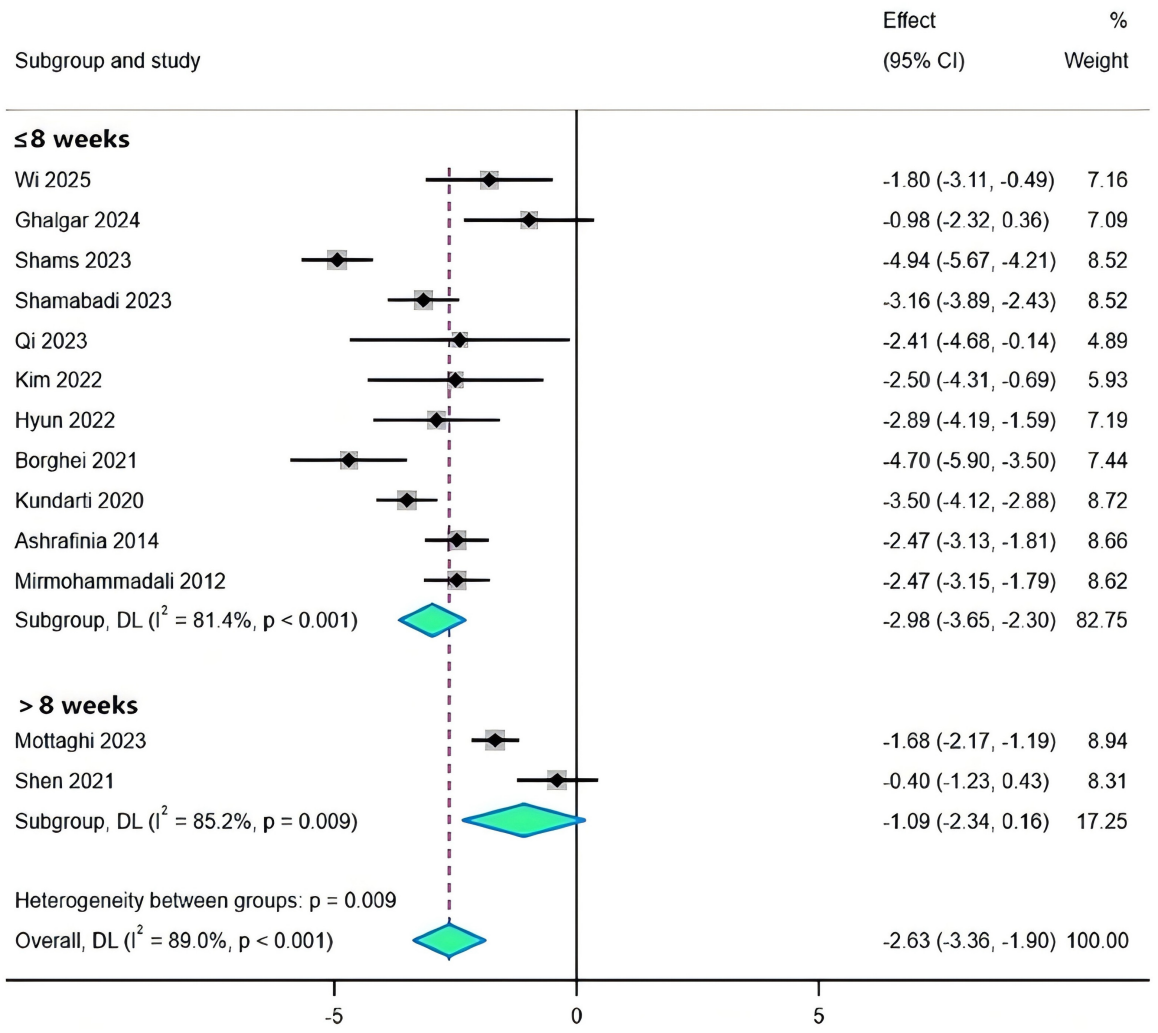
Subgroup analysis by intervention duration.

Subgroup analysis was performed by intervention duration (Figure 9). Based on previous findings that physical activity lasting no more than 8 weeks may be more effective than longer programs for improving sleep disturbances, we used 8 weeks as the cutoff for subgroup analysis (20). The test for subgroup differences showed a highly significant difference between the two duration groups (Q = 6.79, df = 1, *P* < 0.01), suggesting that intervention duration may be an important source of the overall heterogeneity. The pooled results showed that mind–body therapies programs lasting 8 weeks or less significantly improved sleep quality in perinatal women (MD = −2.98, 95% CI −3.65 to −2.30). In contrast, programs lasting more than 8 weeks did not show a statistically significant effect (MD = −1.09, 95% CI −2.34 to 0.16).

**Figure 10:**
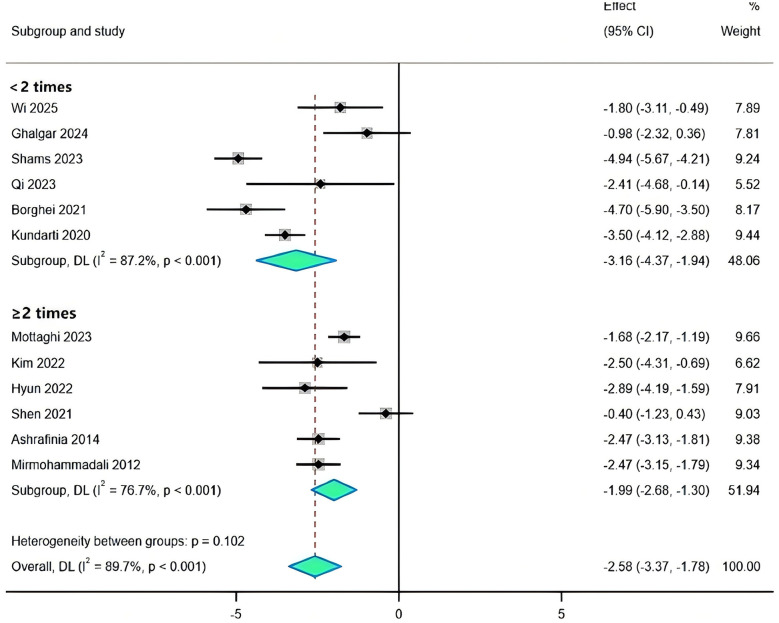
Subgroup analysis by intervention frequency.

Subgroup analysis was performed by intervention frequency (Figure 10). Because the intervention frequency could not be obtained from the study by Shamabadi, that study was excluded from the analysis of intervention frequency (33). Based on previous findings, intervention frequency was divided into 1–2 sessions per week and more than 2 sessions per week (46), intervention frequency was divided into fewer than 2 sessions per week and 2 or more sessions per week. The test for subgroup differences showed no significant difference between intervention frequencies (Q = 2.67, df = 1, *P* > 0.05). The pooled results showed that mind–body therapies significantly improved sleep quality in perinatal women at both frequencies: fewer than 2 sessions per week (MD = −3.16, 95% CI −4.37 to −1.94) and 2 or more sessions per week (MD = −1.99, 95% CI −2.68 to −1.30).

**Figure 11:**
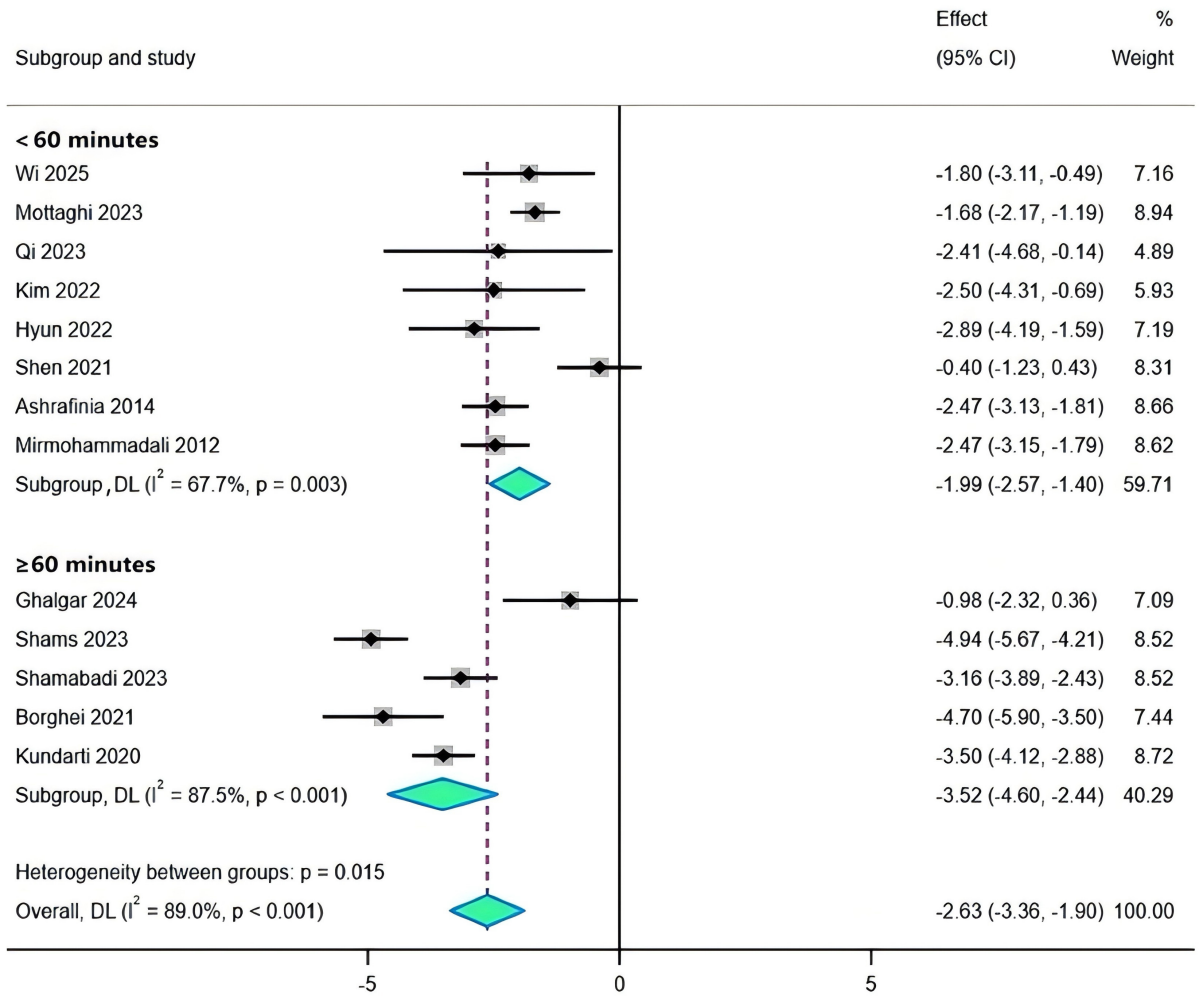
Subgroup analysis by session length.

Subgroup analysis was performed by session length (Figure 11). Previous studies have shown that interventions lasting at least 60 min per session may be more effective for improving sleep disturbances than those lasting less than 60 min. Therefore, 60 min per session was used as the cutoff for subgroup analysis in this study (20). The test for subgroup differences showed a significant difference between session lengths (Q = 5.96, df = 1, *P* < 0.05), suggesting that session length may be an important source of the overall heterogeneity. The pooled results showed that mind–body therapies significantly improved sleep quality in perinatal women whether the session lasted less than 60 minutes (MD = −1.99, 95% CI −2.75 to −1.40) or at least 60 minutes (MD = −3.52, 95% CI −4.60 to −2.44).

The above subgroup analyses showed that study country (*P* < 0.05), intervention duration (*P* < 0.01), and session length (*P* < 0.05) may be the main sources of heterogeneity. In addition, the subgroups with an intervention duration of 8 weeks or less (MD = −2.98) and a session length of at least 60 min (MD = −3.52) showed larger effects on sleep quality improvement, and the corresponding between-subgroup differences reached statistical significance. These findings suggest that these dose-related factors may have potential advantages in improving sleep quality among perinatal women. However, these findings were based on study-level subgroup comparisons and should be interpreted with caution.

### 3.6 Sensitivity test and bias test

#### 3.6.1 Sensitivity test

**Figure 12:**
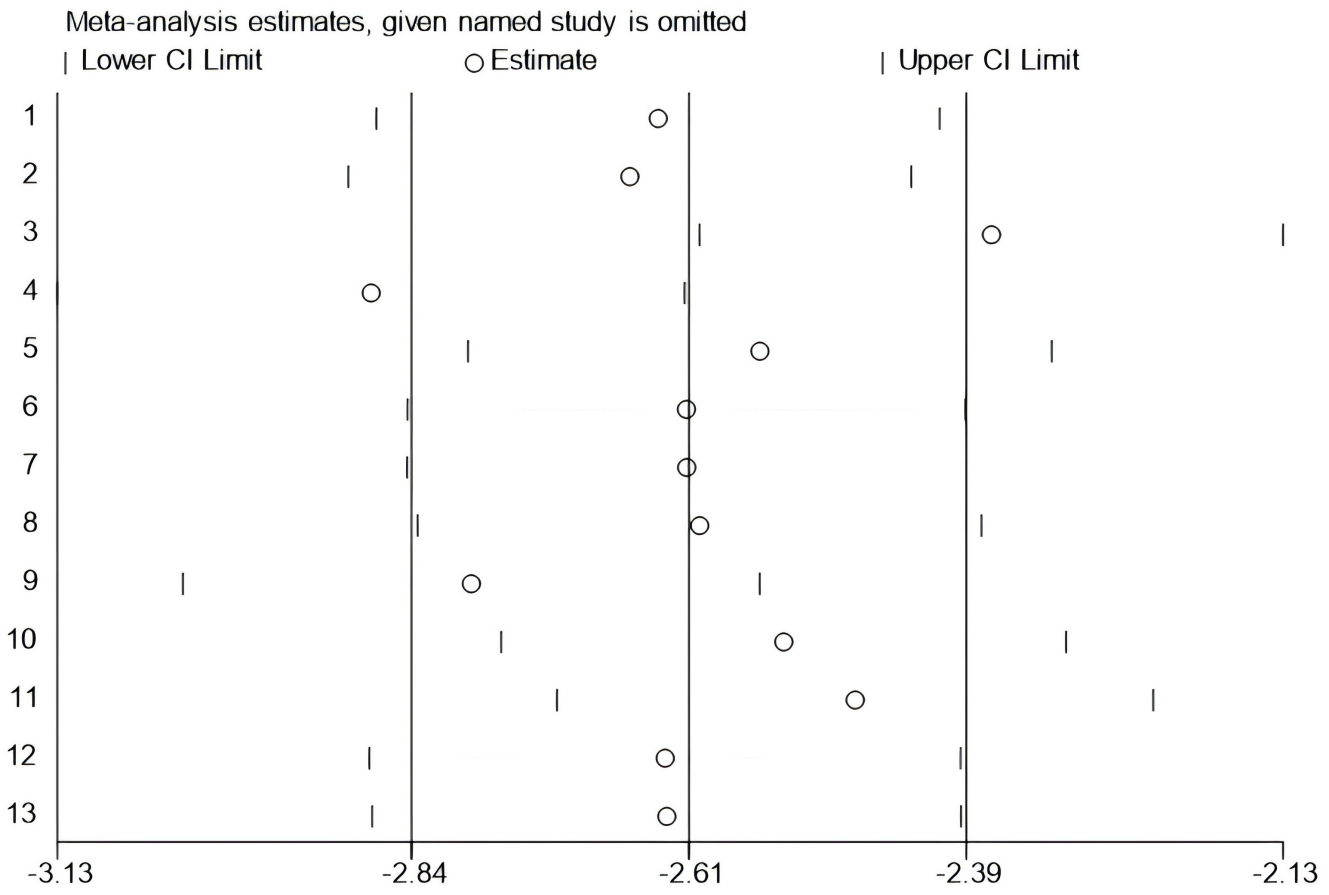
Sensitivity analysis of the effects of mind-body exercise on sleep quality in perinatal women.

To assess the robustness and reliability of the pooled results, we performed a leave-one-out sensitivity analysis by removing each included study in turn. The results are presented as a bubble plot (Figure 12). After exclusion of any single study, the direction of the pooled effect did not change. The point estimates remained negative, and the 95% CI also stayed below zero. These findings were highly consistent with the main analysis. Together, they suggest that the pooled effect was not unduly driven by any individual study and that the overall meta-analytic findings are robust and reliable.

#### 3.6.2 Bias risk test

**Figure 13:**
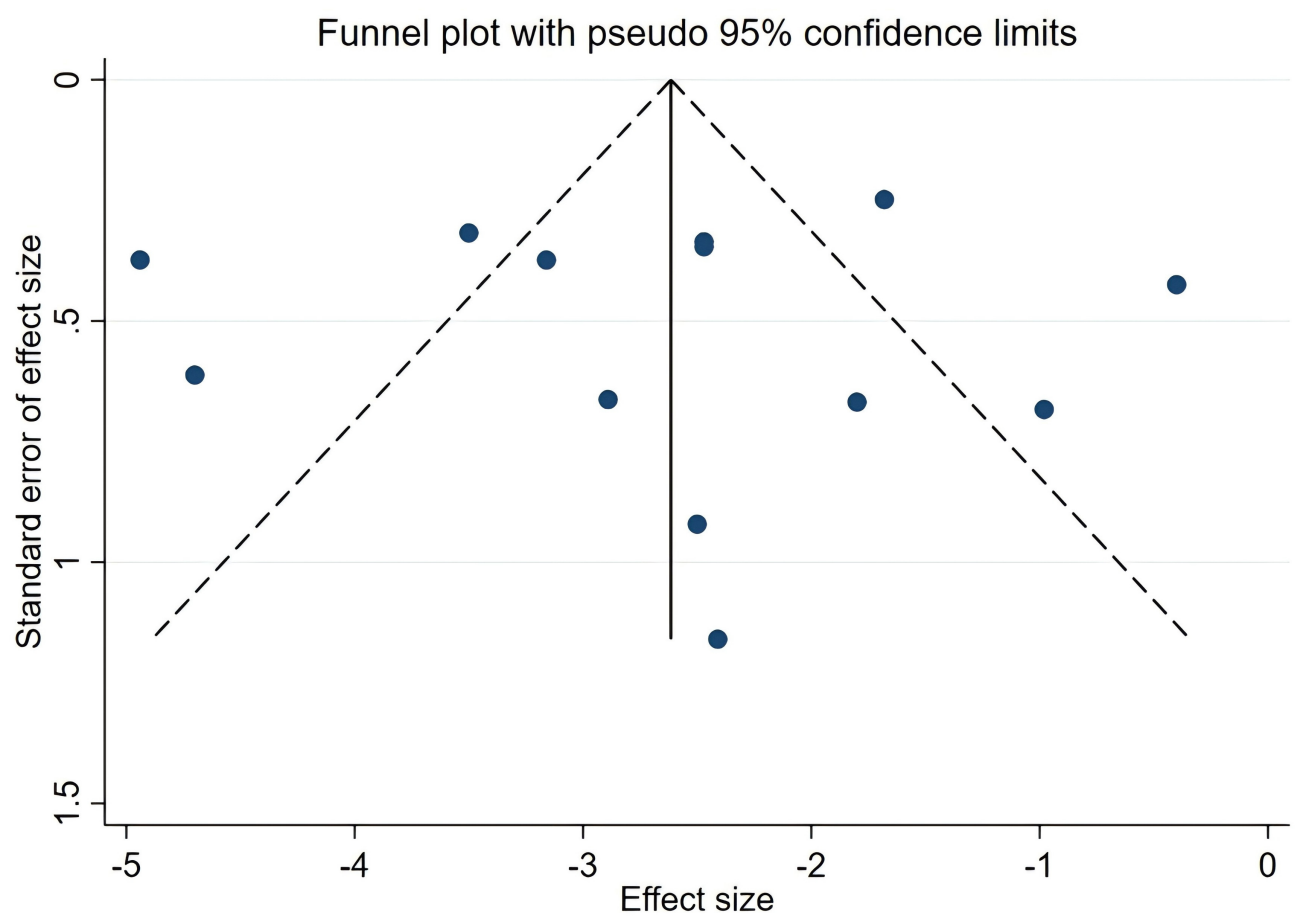
Funnel plot for publication bias in the meta-analysis of mind-body exercise and sleep quality in perinatal women.

Publication bias was assessed using both a funnel plot and Egger's test. The scatter points from the 13 included studies were generally symmetrically distributed, with no obvious visual asymmetry in the funnel plot (Figure 13). Egger's test further showed no statistically significant publication bias (*P* = 0.900). The visual and statistical findings were consistent, suggesting that no substantial publication bias was present among the included studies. Therefore, the reliability of the pooled meta-analytic results is unlikely to have been affected by publication bias.

### 3.7 Quality of evidence assessment

We further assessed the certainty of evidence for the primary outcome, sleep quality represented by the PSQI total score, using the GRADE approach (Table 3). The results showed that the certainty of evidence for this outcome was rated as low. The evidence was downgraded by one level for risk of bias, mainly because most included studies had some concerns and one study had a high risk of bias. The evidence was also downgraded by one level for inconsistency because substantial heterogeneity was observed across studies (I^2^ = 89.0%). No downgrading was applied for indirectness, imprecision, or publication bias, as the population, intervention, comparison, and outcome were consistent with the review question, the effect estimate was negative and the confidence interval did not cross the line of no effect, and funnel plot inspection together with Egger's test did not suggest significant publication bias (*P* = 0.900). Overall, the current evidence suggests that mind–body therapies may improve sleep quality in perinatal women, but the certainty of evidence is low.

**Table 3:** GRADE assessment of the certainty of evidence.

Certainty assessment							No of patients		Effect	Certainty	Importance
No of studies	Study design	Risk of bias	Inconsistency	Indirectness	Imprecision	Other considerations	Intervention	Control	Absolute (95% CI)		
13	Randomized trials	Serious	Serious	Not serious	Not serious	None	397	385	MD 2.63 lower (3.36 lower to 1.90 lower)	⊕⊕○○ Low	IMPORTANT

## 4 Discussion

Based on evidence from 13 randomized controlled trials involving 782 perinatal women, this study systematically evaluated the effects of mind–body therapies on sleep quality. The results showed that, compared with the control group, mind–body therapies significantly improved sleep quality in perinatal women. Although high heterogeneity was observed across studies, sensitivity analysis did not show that any single study had a decisive influence on the overall results, and the publication bias test did not indicate obvious bias. These findings suggest that the main conclusion of this study has a certain degree of robustness. Further subgroup analyses suggested that study country, intervention duration, and session length may partly explain the heterogeneity. Among them, the subgroups with an intervention duration of 8 weeks or less (MD = −2.98) and a session length of at least 60 min (MD = −3.52) showed larger effects on sleep quality improvement, and the corresponding between-subgroup differences reached statistical significance. However, these subgroup findings mainly indicate potential directions of benefit. The current evidence is still insufficient to determine any specific type of mind–body therapies or dose regimen as the “optimal prescription” for sleep intervention in perinatal women.

### 4.1 Effects of mind–body therapies on sleep quality in perinatal women: current evidence and possible mechanisms

The finding that mind–body therapies can significantly improve sleep quality in perinatal women is largely consistent with most previous studies, although some differences remain. Kundarti et al. reported that an 8-week prenatal yoga program, delivered once a week for 90 min per session, significantly improved sleep quality in primiparous women in the second and third trimesters of pregnancy (44). Similarly, Shamabadi found that six mindfulness sessions delivered over 2 months significantly improved sleep quality in women in mid-to-late pregnancy (33). However, it is worth noting that Ghalgar et al. found that an 8-week online mindfulness intervention did not produce a clear improvement in sleep quality among postpartum women (36). We believe that the heterogeneity across studies mainly arose from two key factors. First, the perinatal period includes both pregnancy and the postpartum stage. These two stages differ markedly in hormone levels, physical burden, pain experience, infant-care demands, and patterns of night-time awakening. As a result, the same intervention may not produce the same effect on sleep at different stages (47, 48). Second, most existing studies have focused on a single type of intervention (18, 19), In contrast, our study combined mindfulness, yoga, and Pilates into a broader category of mind–body therapies. We were therefore more concerned with the overall effect of this intervention category than with the effect of any single approach. This also helps explain the slight differences between our findings and those of earlier studies. More importantly, the primary finding of this study aligns with evidence from other populations. Previous systematic reviews have shown that mind–body therapies can improve sleep disturbances in older adults and have positive effects on sleep quality, anxiety, depression, and fatigue in perimenopausal and postmenopausal women (16, 17).

Taken together, the available evidence supports a stable and broadly applicable benefit of mind–body therapies for improving sleep quality in perinatal women. The observed heterogeneity across studies can be reasonably explained by differences in the timing of intervention and the scope of analysis. On this basis, a deeper understanding of how mind–body therapies improve sleep during the perinatal period requires a systematic examination from both physiological and psychological perspectives.

It should be noted that none of the included original studies reported physiological indicators. Therefore, the following discussion of physiological mechanisms is based mainly on theoretical inference. At the physiological level, stress and physical discomfort during pregnancy can easily activate the sympathetic nervous system, triggering stress responses such as increased heart rate, rapid breathing, and muscle tension. These changes directly disrupt the body's relaxed state and may lead to difficulty falling asleep and poorer sleep quality (46). Mind–body therapies, by contrast, may effectively activate parasympathetic activity, which counteracts sympathetic activation. In doing so, it may reduce sympathetic excitability, relieve physical tension, promote relaxation, and lessen excessive arousal, thereby improving sleep quality in perinatal women through physiological regulation (49). At the psychological level, mind–body therapies may improve sleep by regulating the neural pathways between the prefrontal cortex and the limbic system, which is involved in emotional processing. Through this process, it may reshape the structure and function of emotion-related circuits, thereby improving sleep quality (50). It should be noted that the explanations above are still somewhat speculative. Future studies should incorporate multidimensional outcome measures and use mediation analysis to more clearly identify the key mechanisms through which mind–body therapies exert their effects during the perinatal period.

### 4.2 Subgroup differences in the effects of Mind–body therapies on sleep quality in perinatal women

To further explain the high heterogeneity observed in the overall analysis and to explore potential factors influencing the effects of mind–body therapies on sleep quality in perinatal women, we conducted subgroup analyses according to perinatal stage, country, intervention type, intervention duration, intervention frequency, and session length. The results suggested that country, intervention duration, and session length may be the main sources of heterogeneity in this study, whereas the between-subgroup differences for perinatal stage, intervention type, and intervention frequency were not statistically significant. It is worth noting that the subgroup findings may be influenced by both between-subgroup differences and within-subgroup methodological variability. Even within the same subgroup, studies may not be fully consistent in their intervention protocols or study designs. Differences may remain in intervention type, intervention duration, weekly frequency, session length, mode of practice, and participants' perinatal stage. Due to the limited number of included studies, we were unable to conduct more detailed stratified analyses within subgroups. Therefore, the subgroup findings should be interpreted as exploratory and with caution.

We observed that the prevalence and severity of poor sleep quality in perinatal women may increase as pregnancy progresses (8). Therefore, the timing of the intervention may influence the effects of mind–body therapies. However, the number of included studies was limited. Thus, we could not conduct more detailed subgroup analyses according to early, middle, and late pregnancy or different postpartum stages. Instead, we broadly classified the perinatal period into pregnancy and postpartum periods. Our subgroup analysis showed that the pregnancy subgroup had a numerically greater improvement in sleep quality than the postpartum subgroup (MD = −2.81). However, the between-subgroup difference was not statistically significant (*P* > 0.05). This finding suggests that mind–body therapies may improve sleep quality in both pregnant and postpartum women. Sleep problems may differ between pregnancy and the postpartum period. During pregnancy, they are more likely to be related to physiological changes, physical discomfort, and emotional fluctuations (51). In the postpartum period, they may be more strongly influenced by nighttime breastfeeding, infant care, and postpartum adjustment stress (9). Nevertheless, sleep problems in both stages are closely related to increased physiological burden, changes in daily routines, and psychological stress. Mind–body therapies integrate physical activity and psychological regulation. Therefore, they may have positive effects on sleep across different perinatal stages (52). However, sleep problems may vary across early, middle, and late pregnancy and different postpartum stages. Future studies should further compare the effects of mind–body therapies across these stages. This may help identify the most appropriate stage for intervention.

Subgroup analysis by country showed significant between-group differences (*P* < 0.05), suggesting that study location may be an important source of heterogeneity in this review. This variation may be explained by two main factors. First, countries may differ in perinatal care models, health education, adherence to the intervention, and family support structures. All of these factors may influence the actual effectiveness of the intervention (53). Second, the number of included studies was uneven across countries. For example, only two studies were conducted in China, whereas seven were from Iran. Effect estimates in small subgroups are more easily influenced by individual studies and may therefore be less stable. These findings suggest that, when promoting mind–body therapies programs for sleep management in clinical practice, the intervention should be adapted to the local perinatal care system and population characteristics.

Subgroup analysis showed that non-supervised, online-supervised, and offline-supervised interventions all significantly improved sleep quality in perinatal women. However, the between-subgroup difference among the three supervision formats was not statistically significant, suggesting that current evidence is insufficient to demonstrate a clear advantage of any specific supervision format. Previous research has reported that, compared with telephone supervision, face-to-face supervision may improve the quality of exercise implementation through direct guidance, timely feedback, intensity monitoring, and movement correction, leading to better outcomes in functional exercise capacity, quality of life, and physical activity levels (54). However, the findings of the present study are not fully consistent with those results, which may be related to differences in intervention content. In the present study, some online-supervised or non-supervised interventions were mainly mindfulness-based practices. These interventions involved relatively low physical intensity and may have relied less on movement accuracy and on-site correction. After receiving course materials, online reminders, or initial guidance, participants may still have been able to complete the intervention adequately. Therefore, in clinical practice, it may be inappropriate to emphasize the absolute superiority of any single supervision format. Instead, an appropriate supervision format should be selected flexibly according to perinatal women's physical condition, exercise experience, time availability, transportation accessibility, family support, and self-management ability. Finally, given the limited number of studies in each subgroup, the findings regarding supervision format should be interpreted as exploratory and with caution.

Subgroup analysis showed that both the mild sleep disturbance group and the severe sleep disturbance group benefited from mind–body therapies. However, the between-subgroup difference was not statistically significant. Song et al. conducted a network meta-analysis on sleep quality in older adults and stratified participants according to baseline PSQI levels. Their results suggested that responses to non-pharmacological interventions may differ across populations with different baseline sleep quality. Some interventions showed greater improvements in sleep quality among participants with higher baseline PSQI scores (55). This difference may be related to the population characteristics and analytical methods of the present study. The present study focused on perinatal women, whose sleep quality is affected not only by the severity of baseline sleep disturbance, but also by pregnancy-related physiological changes, postpartum caregiving burden, nocturnal awakenings, emotional status, and family support. Therefore, the independent effect of baseline sleep quality on intervention outcomes may be weakened by other perinatal-specific factors (56). In addition, compared with the above network meta-analysis, the present study conducted a conventional subgroup analysis based only on the mean baseline PSQI scores of the included studies. The number of included studies was also limited, which prevented further comparison of the relative effects of different intervention types across different baseline sleep levels. Therefore, the moderating role of baseline sleep quality in the effect of mind–body therapies should be further examined in future studies with larger sample sizes, individual participant data, or more refined meta-analytic approaches.

In terms of intervention type, subgroup analysis showed no significant between-group difference among mindfulness, Pilates, and yoga (*P* > 0.05). This suggests that the current evidence is still insufficient to support a clear advantage for any single form of mind–body therapies. However, judging from the effect sizes, mindfulness (MD = −3.08) showed a relatively stronger trend toward improving sleep. This may be because it acts more directly on worry and intrusive thoughts, reduces anxious anticipation, and lowers cognitive arousal before sleep. A reduction in rumination may also contribute to this effect (57, 58). By contrast, yoga did not show a statistically significant effect. This may be closely related to the fact that only two yoga studies were included, with limited sample sizes and substantial heterogeneity. Therefore, although mindfulness appears to have some potential advantage in improving sleep quality in perinatal women, it cannot yet be regarded as the optimal intervention. More large-sample studies with low heterogeneity and direct comparative designs are still needed.

In terms of training dose, programs lasting 8 weeks or less showed a larger effect than those lasting more than 8 weeks (*P* < 0.01), and sessions lasting 60 min or longer showed a larger effect than sessions lasting less than 60 min (*P* < 0.05). For intervention frequency, the between-subgroup difference between 1–2 sessions per week (MD = −3.16) and more than 2 sessions per week (MD = −1.99) was not statistically significant (P > 0.05). These findings suggest that improvement in sleep quality during the perinatal period may not simply depend on accumulating more training. It may be more closely related to how the training is scheduled over time. Notably, this pattern is not unique to the present study. A recent study on exercise and sleep quality during pregnancy reported a similar trend (20). For perinatal women, multiple real-world burdens, including pregnancy-related discomfort, limited time, differences in family support, and postpartum caregiving demands, may all affect participation and adherence (53). Therefore, when designing future mind–body therapy interventions, intervention duration and session length may be considered as important reference factors, with flexible adjustments according to individual conditions. However, because these findings were mainly derived from study-level subgroup comparisons and the number of included studies was limited, they should be interpreted with caution and cannot yet support definitive recommendations regarding the optimal intervention dose.

### 4.3 Limitations and future directions

Although this study provides evidence for mind–body therapy interventions in perinatal women, several limitations should be noted. First, the overall sample size was small, and some subgroup analyses included only a limited number of studies. In addition, substantial heterogeneity was observed in the main analysis (I^2^ = 89.0%). These factors may have reduced statistical power and limited the stability and interpretability of the pooled estimates. Second, the overall methodological quality of the included studies was relatively low, which may affect the certainty of the evidence. The ROB2 assessment showed that most studies had some concerns, and one study had a high risk of bias. In particular, nine studies did not provide sufficient information on random sequence generation, allocation concealment, or the detailed implementation of random assignment, which may increase the risk of selection bias and affect the accuracy of the estimated intervention effects. Third, the included studies differed in how the interventions were delivered. For example, some used online unsupervised programs, whereas others used face-to-face guided interventions. These differences may have affected the consistency of the results. In addition, methodological heterogeneity may still exist within some subgroups, including differences in intervention type, intervention duration, intervention frequency, session length, mode of practice, and participants' perinatal stage. Due to the limited number of included studies, we were unable to conduct more detailed stratified analyses or meta-regression. Therefore, the stability and interpretability of the subgroup findings may have been affected. Fourth, only the total Pittsburgh Sleep Quality Index score was used as the outcome measure. We did not examine changes in specific dimensions, such as sleep latency, sleep efficiency, or daytime functioning. Objective sleep measures, such as polysomnography, were also not included. As a result, it remains unclear which aspects of sleep quality are most improved by mind–body therapies.

Future studies should report randomization procedures, allocation concealment, blinding, adherence, and outcome data more rigorously, and should use larger samples and more standardized intervention protocols to improve study quality and evidence credibility.

## 5 Clinical significance

This study confirms that mind–body therapy interventions are safe, effective, and feasible non-pharmacological interventions for improving sleep quality during the perinatal period. They may be widely applied in obstetric clinical care and community-based perinatal health services.

## 6 Conclusion

Mind–body therapies are effective non-pharmacological interventions for improving sleep quality in perinatal women and may act as a useful adjunct to clinical sleep health management for this population. However, the certainty of evidence was rated as low according to the GRADE assessment, and the findings were limited by the small sample sizes of the included studies and heterogeneity across studies. More high-quality studies with larger samples are needed to further confirm these results.

## Data Availability

The original contributions presented in the study are included in the article/supplementary material, further inquiries can be directed to the corresponding author.
